# Multi-Feature Adaptive Variational Mode Decomposition for Wearable ECG Devices

**DOI:** 10.3390/bios16050262

**Published:** 2026-05-01

**Authors:** Zixin Chen, Di Wu, Yuanlin Nie, Junwei Zhang, Guanzhou Liu, Feng He, Long Mo, Liming Peng, Chang Zeng, Zhengchun Liu

**Affiliations:** 1 School of Electronic Information, Central South University, Changsha 410083, China; 232212110@csu.edu.cn (Z.C.); 244502015@csu.edu.cn (D.W.); 244512061@csu.edu.cn (Y.N.); 232212115@csu.edu.cn (J.Z.); 244512078@csu.edu.cn (G.L.); 2 School of Mechanical and Electrical Engineering, Central South University, Changsha 410083, China; hefeng84928@163.com; 3 School of Xiangya Medicine, Central South University, Changsha 410083, China; longmo@csu.edu.cn (L.M.); xypengliming@csu.edu.cn (L.P.); echozengchang@csu.edu.cn (C.Z.)

**Keywords:** ECG signal, motion artifact, Variational Mode Decomposition (VMD), wearable devices, adaptive denoising, signal processing

## Abstract

To address the issue of motion artifact interference faced by wearable ECG monitoring devices in dynamic environments, this paper proposes an adaptive motion artifact removal framework based on improved Variational Mode Decomposition (VMD). By designing a parameter self-adjustment mechanism and a multi-feature fusion mode selection strategy, the algorithm’s adaptability to non-stationary ECG signals and noise separation accuracy are enhanced. Experiments on the MIT-BIH Arrhythmia Database demonstrate that the improved VMD algorithm outperforms traditional wavelet transform, Recursive Least Squares (RLS), and conventional VMD methods in multiple performance metrics. Specifically, the signal-to-noise ratio (SNR) is improved by 5.17 dB, the Percentage Root Mean Squared Difference (PRD) is reduced to 49.13%, the correlation coefficient is increased to 0.88, and high real-time processing capability (Real-Time Processing Ratio, RTR = 22.5) is maintained, meeting the low-latency requirements of wearable devices. Moreover, case studies on pathological recordings (e.g., Wolff–Parkinson–White syndrome and third-degree atrioventricular block) reveal that the improved VMD better preserves clinically significant features such as delta waves and dissociated P waves. Furthermore, a downstream arrhythmia classification task using a CWT-CNN classifier achieves 91.67% accuracy on denoised heartbeats, which is 2.67 percentage points higher than that on raw noisy signals (89.00%), confirming the practical benefit of the proposed preprocessing for AI-based diagnosis. This study provides an effective processing solution for improving the signal quality of wearable ECG monitoring.

## 1. Introduction

With the rapid development of flexible electronics technology, the Internet of Things (IoT), and artificial intelligence (AI), continuous health monitoring based on wearable devices has become a research hotspot and industrial trend in the field of digital healthcare [1,2]. As a key physiological parameter for evaluating cardiovascular function, long-term, dynamic, and non-intrusive monitoring of electrocardiogram (ECG) signals holds significant clinical value for the early warning, diagnosis, and rehabilitation assessment of cardiovascular diseases such as arrhythmia and myocardial ischemia [3]. According to statistics from the World Health Organization (WHO), cardiovascular diseases (CVDs) have become the leading cause of death globally, with annual deaths reaching 17.9 million, accounting for 32% of the global total deaths [4]. Wearable ECG monitoring devices can achieve 24/7 continuous monitoring, providing an unprecedented technical means for the early detection of cardiovascular events. However, during the use of wearable devices (e.g., smartwatches and chest-worn monitors), motion-related noise caused by daily human activities, physical exercise, and other movements—including baseline drift, electromyographic interference, and limb motion artifacts—is unavoidable. These noises exhibit variable forms and their frequency spectra highly overlap with ECG signals (0.1–10 Hz), severely degrading ECG signal quality, leading to increased R-wave missed detection and false detection rates, and affecting advanced diagnoses such as subsequent ST-segment analysis, QT interval measurement, and arrhythmia classification [5,6]. Studies have shown that under moderate-intensity exercise conditions, the signal quality of traditional ECG devices can decrease by more than 60%, greatly limiting their progress from consumer-grade to medical-grade applications [7,8]. Therefore, designing an efficient, robust motion artifact removal algorithm suitable for the resource-constrained environment of wearable devices has become a core challenge in promoting the clinical application of wearable ECG monitoring technology.

Currently, research on motion artifact removal for wearable ECG mainly focuses on three technical routes. Time-frequency transform-based methods, represented by wavelet transform, separate noise through multiresolution analysis and threshold processing [9]. However, fixed wavelet basis functions and threshold strategies are difficult to adapt to the non-stationary characteristics of ECG signals, often resulting in distortion of QRS complex features [10,11]. Adaptive filter-based methods such as Recursive Least Squares (RLS) filters use reference signals from accelerometers for noise cancellation [12,13], but their performance is highly dependent on the linear correlation between reference signals and artifacts, leading to limited effectiveness in sudden, non-stationary motion scenarios [14,15]. Among signal decomposition-based methods, Variational Mode Decomposition (VMD) has attracted much attention due to its solid mathematical foundation and excellent mode separation capability [16]. Nevertheless, traditional VMD methods have two key bottlenecks: first, decomposition parameters (number of modes K, bandwidth constraint α) need to be preset based on experience, lacking adaptability; second, the selection of Intrinsic Mode Functions (IMFs) lacks objective criteria, and fixed selection strategies are mostly adopted, making it difficult to adapt to complex noise environments [17,18].

To address the above challenges, this paper proposes an adaptive VMD motion artifact removal framework for wearable devices [19,20]. The framework achieves dual innovations in theory and methodology: first, an adaptive parameter initialization mechanism based on signal length is proposed to dynamically adjust VMD parameters according to data length, enhancing the algorithm’s adaptability to signals of different durations; second, an intelligent multi-feature fusion IMF selection strategy is designed [21]. By comprehensively evaluating the spectral characteristics (QRS band power ratio), statistical characteristics (kurtosis), and correlation with the original signal of each IMF, a weighted scoring model is constructed to realize automatic and interpretable separation of signal and noise components. To verify the effectiveness of the proposed framework, a mixed noise environment simulating real motion scenarios is constructed on a public dataset, and comprehensive performance comparison and real-time evaluation are conducted with five mainstream algorithms. Experimental results show that the improved VMD algorithm proposed in this paper is significantly superior to existing methods in denoising performance, waveform fidelity, and algorithm robustness, while meeting the real-time processing requirements of wearable devices. Furthermore, a subsequent arrhythmia classification experiment demonstrates that the denoised signals lead to a 91.67% detection accuracy, outperforming raw noisy signals (89.00%) by 2.67 percentage points. This study provides an effective technical solution for improving the clinical availability of wearable ECG monitoring data [22].

## 2. Methods

### 2.1. Dataset and Preprocessing

To comprehensively evaluate the performance and robustness of the algorithm, the MIT-BIH Arrhythmia Database is adopted as the experimental benchmark. This database contains 48 half-hour dual-channel dynamic electrocardiogram records with a sampling rate of 360 Hz, covering various types of arrhythmias, and is a standard dataset for evaluating ECG signal processing algorithms. To simulate the noise challenges faced by wearable devices in dynamic environments, artificial motion artifacts, including baseline drift, electromyographic interference, motion artifacts, and mixed noise, are added to clean ECG signals, and the signal-to-noise ratio (SNR) level of the added noise is set. The added motion artifacts have typical characteristics such as large amplitude, wide frequency overlap with ECG signals, and strong non-stationarity, which can effectively simulate noise interference in real dynamic environments.

**Figure 1:**
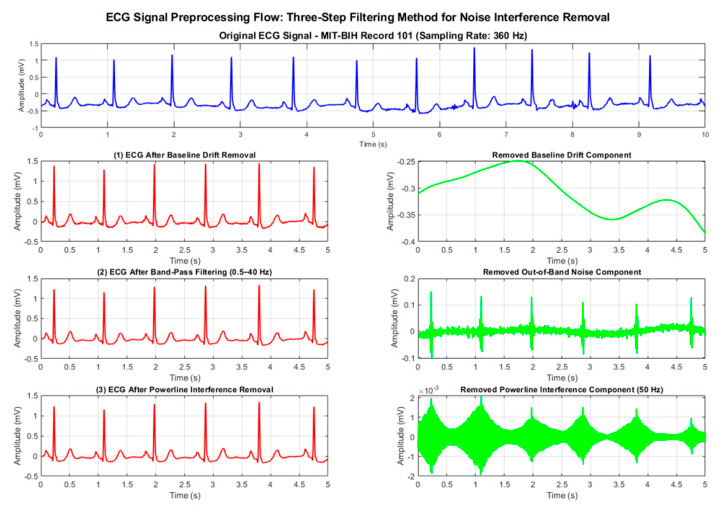
ECG signal preprocessing and removed noise.

As shown in Figure 1, the signal preprocessing process includes the following steps: (1) A second-order Butterworth high-pass filter (cutoff frequency 0.5 Hz) is used to eliminate baseline drift; (2) a band-pass filter (0.5–40 Hz) is applied to retain the effective frequency band of ECG; (3) a notch filter (50 Hz) is used to eliminate power line interference.

To construct a controllable mixed noise experimental environment, three typical artificial motion artifacts are linearly superimposed on the preprocessed “clean” ECG signal to generate a noisy signal:

(1) $$s_{noisy}\left(t\right)=s_{clean}\left(t\right)+\Sigma_{i=1}^{3}\beta_{i}\cdot n_{i}\left(t\right)$$

Among them $n_{1}\left(t\right)$ is low-frequency baseline drift (0.05–0.5 Hz), $n_{2}\left(t\right)$ is sudden electromyographic interference (broadband Gaussian noise superimposed with random spikes), and $n_{3}\left(t\right)$ is a quasi-periodic limb motion artifact (1–5 Hz). By adjusting the noise component weights $\beta_{i}$ and SNR, mixed noise scenarios of different intensities and types are simulated to ensure the comprehensiveness of the algorithm evaluation.

### 2.2. Design of Improved Variational Mode Decomposition (VMD) Algorithm

Traditional VMD algorithms have defects of fixed decomposition parameters and subjective IMF component selection, making it difficult to adapt to the non-stationary characteristics and complex noise environments of wearable ECG signals. This paper proposes a multi-feature adaptive VMD algorithm, which improves the traditional VMD from two dimensions—parameter-adaptive adjustment and intelligent IMF selection—realizing effective separation of ECG signals and motion artifacts.

#### 2.2.1. Basic Principle of VMD

Variational Mode Decomposition is an adaptive signal decomposition method based on variational problem solving. Its core is to decompose a signal into K Intrinsic Mode Functions (IMFs) $u_{\kappa }\left(t\right)(\kappa =1,2,\ldots ,K)$ with specific bandwidths. The variational problem of VMD is constructed as follows:

(2) $$\left\{\begin{array}{c} {min}_{\left\{u_{k}\right\},\left\{w_{k}\right\}}\sum_{k=1}^{K}∥\partial_{t}\left[\left(\delta \left(t\right)+\frac{j}{\pi t}\right)*u_{k}\left(t\right)\right]e^{-jwkt}{∥}_{2}^{2}\\ s.t.\sum_{k=1}^{K}u_{k}\left(t\right)=x\left(t\right) \end{array}\right.$$

where $w_{k}$ is the center frequency of the k-th IMF, $\delta \left(t\right)$ is the Dirac delta function, and * denotes the convolution operation. Here, α is the bandwidth penalty factor. It controls the bandwidth of each mode: a larger α enforces a narrower frequency band. In the context of VMD, α is dimensionless (a relative weight) and is typically set empirically or adaptively.α is a dimensionless regularization parameter that balances the bandwidth constraint; its value is chosen based on signal characteristics (see Equation (3)). By introducing the Lagrange multiplier $\lambda \left(t\right)$ and penalty factor α, the constrained variational problem is transformed into an unconstrained one, and the Alternating Direction Method of Multipliers (ADMM) is applied iteratively to obtain each IMF component and center frequency.

Traditional VMD methods have two key defects: (1) decomposition parameters K (number of modes) and α (bandwidth penalty factor) need to be preset based on experience; (2) the selection of IMF components lacks objective criteria. Aiming at the wearable ECG monitoring scenario, this paper proposes a multi-feature adaptive improved VMD framework [23].

#### 2.2.2. VMD Parameter-Adaptive Adjustment Based on Signal Length

In traditional VMD algorithms, the number of modes K and bandwidth penalty factor α are usually set based on experience (e.g., fixed K = 8, α = 2000). For short-duration ECG signals (such as real-time data blocks from wearable devices with lengths below 500 points), an excessive number of modes may lead to over-decomposition, whereas long-duration signals may suffer from under-decomposition.

To address this issue, this paper proposes a parameter-adaptive strategy that adjusts both K and α according to the signal length N:

(3) $$\left\{\begin{array}{c} K=\left\{\begin{array}{c} 6,N<1000\\ 8,N\geq 1000 \end{array}\right.\\ \alpha =\left\{\begin{array}{c} 1000,N<1000\\ 1500,N\geq 1000 \end{array}\right. \end{array}\right.$$

For short signals (N < 1000), smaller values of K and α are adopted to avoid distortion caused by over-decomposition; for longer signals (N ≥ 1000), larger values ensure adequate separation of noise components.

#### 2.2.3. Noise-Level Awareness and Dynamic Adjustment of α

To further enhance the algorithm’s adaptability to varying noise intensities, a noise-level awareness mechanism is introduced. First, the noise strength is estimated as:

(4) $$\sigma_{noise}=\frac{median\left(\left|x(t)\right|\right)}{0.6745}$$

where x(t) denotes the input ECG signal. If $\sigma_{noise}>\theta$ (with θ=0.5×std(x(t)) set as a threshold), the signal is considered to have a high noise level, and the bandwidth penalty factor α is adjusted accordingly.

(5) $$\alpha_{adjusted}=\alpha \times [1+{log}_{2}\left(1+\frac{\sigma_{noise}}{\theta }\right)]$$

This mechanism automatically tightens the bandwidth constraint among modes under strong noise conditions, thereby improving noise suppression, while retaining the original α for normal noise levels to preserve signal details.

#### 2.2.4. Rationale for Selecting K, α, and N

The strategy of adapting K and α based on signal length N (Equation (3)) is motivated by the following considerations: short-duration signals are prone to over-decomposition if too many modes are used, hence a smaller K = 6 and α = 1000 are chosen; for longer signals, a larger K = 8 and α = 1500 (or higher) ensure sufficient separation of noise components. Experiments confirm that this length-dependent parameterization effectively avoids both over-decomposition and under-decomposition across varying signal durations, while the noise-aware adjustment of α (Equation (5)) provides an additional layer of robustness in high-noise scenarios.

#### 2.2.5. Multi-Feature Fusion-Based Intelligent IMF Selection Strategy

Traditional VMD often fixes the first several IMFs as signal components, lacking a quantitative basis. The improved VMD algorithm proposed in this paper constructs a multi-feature fusion IMF scoring model, realizing intelligent and adaptive separation of signal and noise components by comprehensively evaluating the spectral, statistical, and correlation features of each IMF.

For the k-th IMF component $u_{k}\left(t\right)$, the following features are extracted and normalized to the interval [0, 1]:

1. Spectral characteristic features

Power spectrum estimation (Welch method) is used to calculate the power spectrum of $u_{k}\left(t\right)$. Let $P_{kk}(f)$ denote the power spectral density of the k-th IMF component $u_{k}\left(t\right)$, estimated using Welch’s averaged periodogram method with a Hamming window and 50% overlap. The QRS band power ratio and noise band power ratio are then defined as:

(6) $$S_{qrs}=\frac{\sum_{f\in \left[5,20\right]}P_{kk}(f)}{\sum_{f\in \left[0,fs/2\right]}P_{kk}(f)}$$

(7) $$S_{noise}=1-\frac{\sum_{f\in \left[0,1\right]\cup \left[30,60\right]}P_{kk}(f)}{\sum_{f\in \left[0,fs/2\right]}P_{kk}(f)}$$

where fs is the sampling rate, [5, 20] Hz is the main frequency band of the QRS complex of ECG signals, and [0, 1] Hz and [30, 60] Hz are the main frequency bands of motion artifacts and electromyographic interference. A larger $S_{qrs}$ and $S_{noise}$ indicate that the IMF contains more effective ECG signals.

2. Statistical characteristic features

The kurtosis $K_{kurt}$ of $u_{k}\left(t\right)$ is calculated to reflect the distribution characteristics of the signal. Effective ECG signals have kurtosis deviating from the normal distribution ($K_{kurt}$ = 3), while noise components are close to the normal distribution. The kurtosis score $S_{kurt}$ is defined as:

(8) $$S_{kurt}=\frac{1}{1+|K_{kurt}-3|}$$

3. Correlation features

The Pearson correlation coefficient r between $u_{k}\left(t\right)$ and the original ECG signal $x\left(t\right)$ is calculated, and the correlation score $S_{corr}=\left|r\right|$ is defined. A higher correlation coefficient indicates a stronger correlation between the IMF and the original ECG signal.

Based on the above features, a comprehensive IMF scoring model is constructed:

(9) $$S_{k}=0.4\cdot S_{qrs}+0.3\cdot S_{noise}+0.2\cdot S_{corr}+0.1\cdot S_{kurt}$$

where 0.4, 0.3, 0.2, and 0.1 are feature weights determined by the analytic hierarchy process (AHP), emphasizing the dominant role of spectral characteristics. Specifically, a pairwise comparison matrix was constructed based on the relative importance of the four features (spectral QRS power ratio, noise band power ratio, correlation with original signal, and kurtosis) for ECG signal quality assessment. The resulting weights were derived from the principal eigenvector of the matrix, with a consistency ratio below 0.1, confirming the reliability of the AHP result. Thus, the weights are not arbitrary but are systematically obtained through a multi-criteria decision-making process. IMF selection is performed according to the comprehensive score, and the intelligent IMF selection process is as follows:

4. Scoring and sorting: Calculate the comprehensive score $s_{k}$ of all K IMFs and sort them in descending order to obtain the sorting index $\left\{i_{1},i_{2},\ldots ,i_{K}\right\}$.
5. Adaptive threshold selection:

- Calculate the threshold $s_{th}=max(0.15,median\left(s_{k}\right)\times 0.8)$. This design combines an absolute threshold (0.15) and a relative threshold (80% of the median), enhancing the algorithm’s adaptability to different signals.
- Select IMFs with scores $S_{k}\geq s_{th}$ as signal components.

6. as signal components:

- Floor mechanism: If the number of selected IMFs is less than 2, forcibly select the top 3 (or all if K < 3) with the highest scores.
- Ceiling mechanism: If the number of selected IMFs exceeds 4, only retain the top 4 with the highest scores. This constraint avoids the signal being contaminated by too many noise IMFs and improves denoising stability.

7. Signal reconstruction and post-processing:

- Sum the selected IMF components to obtain the preliminary denoised signal $\hat{x}\left(t\right)=\Sigma_{k=\Omega }u_{k}(t)$, where Ω is the index set of selected IMFs.
- Final smoothing: To balance the denoising effect and waveform fidelity, post-processing is performed on $\hat{x}\left(t\right)$. A Savitzky–Golay filter is employed for smoothing. The filter window length is adaptively set to approximately 10 ms (round(0.01 × fs)) and ensured to be odd, with a polynomial order of 2. This filter effectively suppresses high-frequency residual noise while better preserving sharp features such as QRS complexes, thereby minimizing the risk of distorting clinically significant waveform characteristics.

This strategy achieves high-precision and robust separation of signal and noise IMFs through multi-dimensional quantitative evaluation and an adaptive decision-making mechanism.

## 3. Experiments

### 3.1. Experimental Setup

Experiments are conducted on the MATLAB R2024b platform (MathWorks, Natick, MA, USA) with a hardware configuration of an Intel Core i7-11800H processor and 16 GB of RAM (Intel Corporation, Santa Clara, CA, USA). Comparative algorithms include traditional wavelet threshold denoising (traditional wavelet), traditional Recursive Least Squares filtering (traditional RLS), traditional fixed-parameter VMD (traditional VMD), adaptive threshold wavelet denoising with QRS complex protection (improved wavelet), and multi-reference signal adaptive RLS filtering (improved RLS). All algorithms use the same data preprocessing flow and are evaluated under a mixed noise environment (SNR = 1 dB).

### 3.2. Evaluation Metrics

1. Signal-to-Noise Ratio (SNR)

SNR is used to measure the ratio of effective components to noise components in a signal. SNR improvement reflects the degree of noise removal by the denoising algorithm, which is calculated as the difference between the SNR of the denoised signal and the SNR of the noisy signal.

First, define the SNR of the noisy signal (${SNR}_{raw})$:

(10) $${SNR}_{raw}=10{log}_{10}(\frac{\sum_{n-1}^{N}x_{clean}(n{)}^{2}}{\sum_{n-1}^{N}{(x}_{noisy}\left(n\right)-x_{clean}(n{))}^{2}})$$

where $x_{clean}\left(n\right)$ is the original clean ECG signal, $x_{noisy}\left(n\right)$ is the ECG signal with motion artifacts, and N is the signal length.

Then define the SNR of the denoised signal (${SNR}_{denoised}$):

(11) $${SNR}_{denoised}=10{log}_{10}(\frac{\sum_{n-1}^{N}x_{clean}(n{)}^{2}}{\sum_{n-1}^{N}{(x}_{denoised}\left(n\right)-x_{clean}(n{))}^{2}})$$

where $x_{denoised}\left(n\right)$ is the ECG signal denoised by the algorithm.

(12) $$\Delta SNR={SNR}_{denoised}-{SNR}_{raw}$$

A larger ΔSNR indicates a better noise removal effect of the algorithm.

2. Mean Squared Error (MSE)

MSE measures the overall deviation between the denoised signal and the clean signal, reflecting the overall distortion degree of the signal:

(13) $$MSE=\frac{1}{N}\sum_{n-1}^{N}{(x}_{denoised}\left(n\right)-x_{clean}(n{))}^{2}$$

A smaller MSE indicates a smaller deviation between the denoised signal and the clean signal and better algorithm performance.

3. Root Mean Squared Error (RMSE)

The square root of the mean squared error, having the same dimension as the original signal, reflects the average amplitude of the error.

(14) $$RMSE=\sqrt{MSE}=\sqrt{\frac{1}{N}\sum_{n-1}^{N}{(x}_{denoised}\left(n\right)-x_{clean}(n{))}^{2}}$$

4. Percentage Root Mean Squared Difference (PRD)

The ratio of the root mean squared error to the square root of the energy of the clean signal, expressed as a percentage, is used to compare errors of signals with different amplitudes.

(15) $$PRD=\sqrt{\frac{\sum_{n-1}^{N}{(x}_{denoised}\left(n\right)-x_{clean}(n{)}^{2}}{\sum_{n-1}^{N}x_{clean}(n{)}^{2}}}\times 100\%$$

A smaller PRD indicates a smaller relative error of the denoised signal and higher signal fidelity of the algorithm.

5. Correlation Coefficient (ρ)

The Pearson correlation coefficient between the denoised signal and the clean signal reflects the similarity in waveform shape between the two.

(16) $$\rho =\frac{\sum_{n-1}^{N}{(x}_{denoised}\left(n\right)-{{\bar{x}}_{denoised})(x}_{clean}\left(n\right)-{\bar{x}}_{clean})}{\sqrt{\sum_{n-1}^{N}{(x}_{denoised}\left(n\right)-{\bar{x}}_{denoised}{)}^{2}\;\sum_{n-1}^{N}x_{clean}(n)-{\bar{x}}_{clean}{)}^{2}}}$$

where ${\bar{x}}_{clean}$ and ${\bar{x}}_{denoised}$ are the means of the clean signal and the denoised signal, respectively.

A ρ closer to 1 indicates that the algorithm retains more morphological features of the ECG signal (such as QRS complexes) during denoising.

6. Real-Time Processing Ratio (RTR)

RTR evaluates the computational feasibility of the algorithm on wearable devices, defined as the ratio of the signal duration to the actual processing time of the algorithm:

(17) $$RTR=\frac{T_{signal}}{T_{processing}}$$

where $T_{signal}$ is the duration of the ECG signal to be processed (unit: seconds) and $T_{processing}$ is the actual time consumed by the algorithm to process the signal (unit: seconds). If RTR>1, it indicates that the algorithm processing speed is faster than the signal acquisition speed, meeting the real-time processing requirements of wearable devices; a larger RTR indicates better real-time performance of the algorithm.

### 3.3. Experimental Results and Analysis

**Figure 2:**
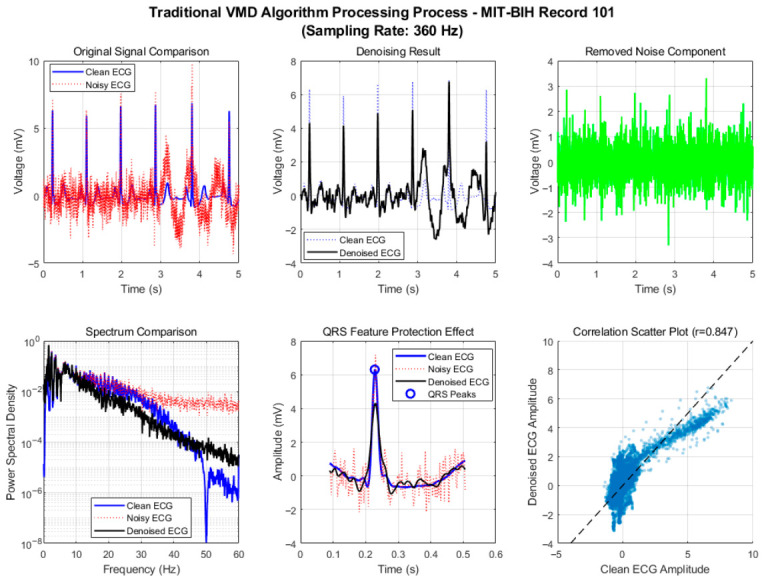
Motion artifact removal process of traditional VMD algorithm.

**Figure 3:**
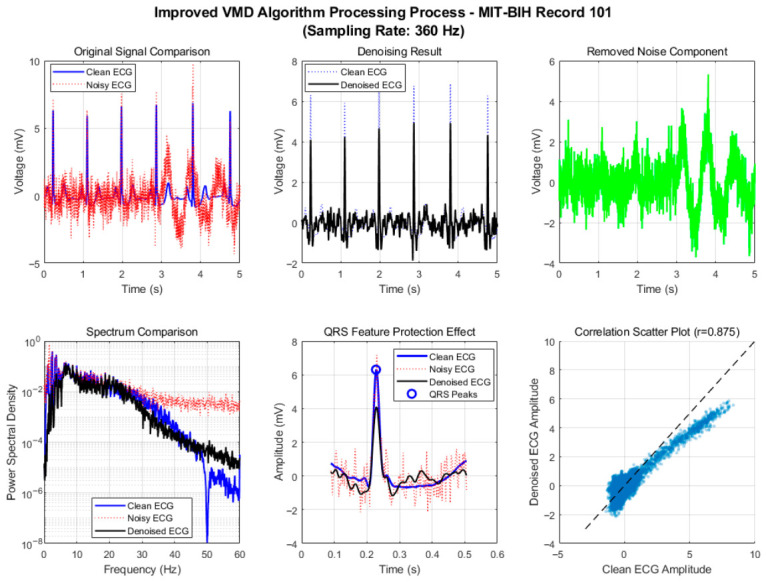
Motion artifact removal process of improved VMD algorithm.

To verify the effectiveness of the proposed adaptive framework and improved algorithm, we conducted a comprehensive comparison of six denoising algorithms using a 60 s segment from MIT-BIH Record 101. After standard preprocessing (baseline removal, band-pass filtering, and powerline interference elimination), mixed motion artifacts were added to the clean ECG signal under a low signal-to-noise ratio (SNR = 1 dB) to simulate challenging dynamic conditions. The algorithms were evaluated across three dimensions—denoising performance, signal fidelity, and computational efficiency—with their processing flows illustrated in Figure 2 and Figure 3.

As shown in Figure 2 and Figure 3, the traditional VMD algorithm realizes the separation of signals and noise through modal decomposition. Thanks to its adaptive spectral decomposition characteristics, its performance is significantly better than that of traditional wavelet and RLS algorithms, but it still has the defects of fixed parameters and subjective selection strategies. The spectral comparison results show that the spectral shapes of the clean ECG and the denoised ECG in the range of 5~45 Hz are relatively close, and the energy distribution of the 5~20 Hz band dominated by QRS complexes is basically consistent, indicating that the algorithm can effectively retain the core frequency components of the ECG signal. In the QRS feature protection effect diagram, the R-peak amplitude and waveform morphology are well preserved, confirming the natural advantage of the modal decomposition method in signal fidelity.

The correlation coefficient of the correlation scatter plot reaches 0.847, reflecting the high similarity between the denoised signal and the original signal, further verifying the waveform fidelity of the algorithm. However, detailed analysis in the QRS band (5~20 Hz) finds that there is a slight attenuation of spectral energy in the 15~20 Hz band, indicating that the fixed IMF selection strategy of the traditional VMD algorithm (summing the first 4 IMF components) lacks pertinence and fails to completely retain the high-frequency detailed components of QRS complexes. In addition, the empirical setting of the number of modes K and bandwidth constraint α leads to residual noise when the algorithm processes high-frequency electromyographic interference, affecting the overall purity of the signal.

**Figure 4:**
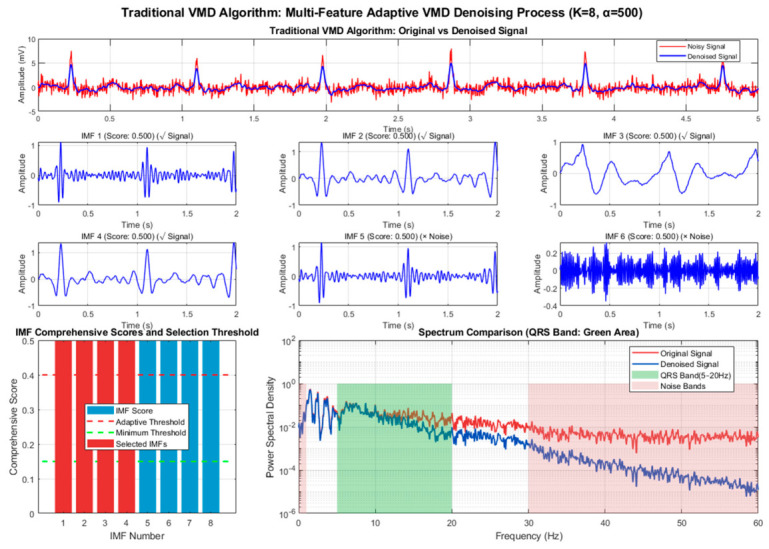
Detailed multi-feature adaptive denoising process of traditional VMD algorithm.

**Figure 5:**
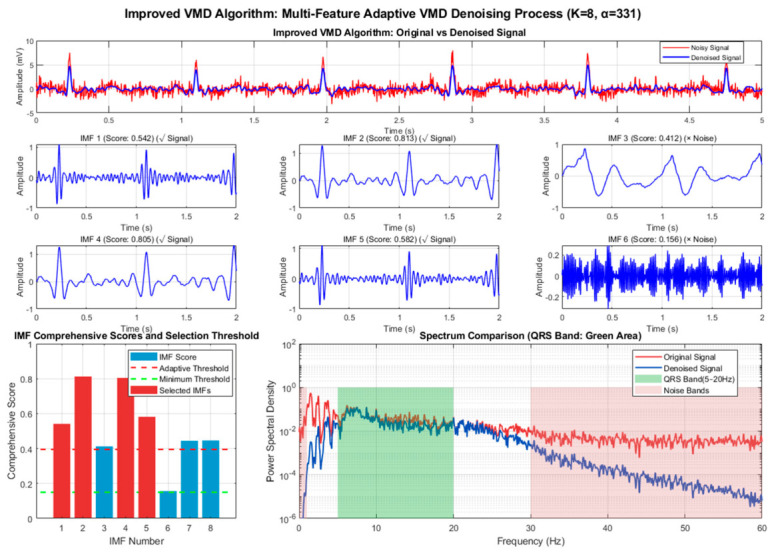
Detailed multi-feature adaptive denoising process of improved VMD algorithm.

As shown in Figure 4 and Figure 5, the improved VMD algorithm achieves the optimal balance between denoising performance and waveform fidelity through parameter-adaptive adjustment and multi-feature fusion-based intelligent IMF selection strategy, and all indicators are optimal. In terms of denoising results, the time-domain waveform shows that the denoised ECG almost completely coincides with the clean signal; baseline drift, electromyographic interference, and motion artifacts are effectively filtered out, and the signal purity is significantly improved. In the spectral comparison results, the spectral energy distribution in the range of 5~45 Hz is highly consistent with that of the clean ECG, especially the spectral curves of the QRS band (5~20 Hz) almost completely overlap, indicating that the algorithm can accurately separate signal and noise components while completely retaining the core frequency characteristics of the ECG signal.

The QRS feature protection effect is particularly prominent. The R-peak amplitude attenuation is small, and the sharp features and time positions of the waveform match well, providing a reliable data basis for subsequent clinical applications such as R-wave detection and ST-segment analysis. This advantage stems from the algorithm’s assignment of 40% weight to the QRS band power ratio in the IMF scoring model, ensuring that the IMF components dominated by QRS complexes are preferentially selected. The correlation coefficient of the correlation scatter plot reaches 0.875, the highest among all algorithms, confirming the high similarity between the denoised signal and the original signal, reflecting the undistorted retention of signal amplitude characteristics by the algorithm.

**Figure 6:**
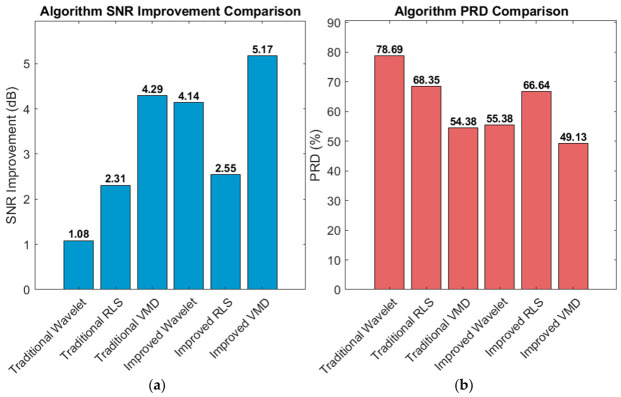
Performance comparison of six denoising algorithms on MIT-BIH Record 101 under mixed motion artifacts (input SNR = 1 dB). (**a**) SNR improvement comparison; (**b**) PRD comparison. The improved VMD achieves the highest ΔSNR (5.17 dB) and the lowest PRD (49.13%), indicating superior noise suppression and waveform fidelity.

**Figure 7:**
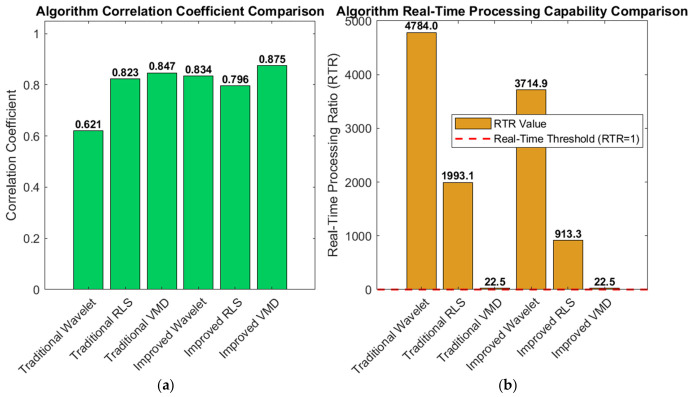
(**a**) Correlation coefficient comparison; (**b**) real-time processing capability comparison. All algorithms meet real-time requirements (RTR > 1). The improved VMD achieves the highest correlation (0.875) while maintaining an RTR of approximately 22.5, demonstrating a good balance between denoising performance and computational efficiency.

Figure 6 and Figure 7 below show the statistical results for different algorithms using MIT-BIH Record 101 as an example.

In terms of denoising performance, VMD-based algorithms have natural advantages in non-stationary noise environments due to their adaptive frequency band decomposition characteristics. Wavelet-based algorithms have high computational efficiency but are prone to QRS feature loss in complex noise environments; RLS-based algorithms are highly dependent on the quality of reference signals and have limited performance in dynamic mixed noise scenarios [24]. While maintaining high real-time processing capability (RTR = 22.5), the improved VMD achieves comprehensive optimal performance with an SNR improvement of 5.17 dB, a PRD reduced to 49.13%, and a correlation coefficient of 0.875, which well balances denoising effect, waveform fidelity, and computational efficiency, meeting the requirements of wearable devices for low-latency and high-fidelity ECG signal processing [25].

In summary, the improved VMD algorithm has the best denoising effect. In terms of denoising capability, the SNR improvement index shows a clear gradient: improved VMD (5.17 dB) > traditional VMD (4.29 dB) > improved wavelet (4.14 dB) > improved RLS (2.55 dB) > traditional RLS (2.31 dB) > traditional wavelet (1.08 dB). Thanks to their adaptive decomposition characteristics, VMD-based algorithms show natural advantages in complex noise environments; in particular, the improved VMD improves SNR by 20.5% compared with the traditional VMD through the multi-feature fusion-based intelligent IMF selection strategy, verifying the effectiveness of the intelligent selection mechanism. The signal fidelity indicators (PRD and correlation coefficient) are consistent with the trend of SNR improvement. The improved VMD has the lowest PRD (49.13%) and the highest correlation coefficient (0.875), indicating that it retains the morphological characteristics of the original ECG to the greatest extent while removing noise.

QRS feature protection is an important dimension for evaluating ECG denoising algorithms. Traditional algorithms generally have the problem of excessive smoothing of QRS complexes: traditional wavelets lead to a significant reduction in R-wave amplitude, traditional RLS causes distortion of QRS complexes during the convergence stage, and traditional VMD may lose high-frequency QRS information due to fixed IMF selection. Improved algorithms effectively alleviate this problem through specialized mechanisms: the improved wavelet detects 212 QRS complexes and sets an 80 ms protection window, mixing 20% of the original signal in the protection area; the improved RLS protects 214 QRS complexes and adopts 40% original signal mixing; the improved VMD assigns 40% weight to the QRS band power ratio (5–20 Hz) feature in the IMF score to ensure that QRS components are preferentially selected. Spectral characteristics and time-domain waveform comparison show that the sharp features of the QRS complexes of the improved algorithms are better preserved, the R-wave amplitude is close to the original signal, and the frequency components are effectively protected.

Spectral characteristics and time-domain waveform comparison show that the sharp features of QRS complexes of the improved algorithms are better preserved, the R-wave amplitude is close to the original signal, and the frequency components are effectively protected. Traditional wavelets excessively reduce the energy of the 5–20 Hz QRS band, leading to attenuation of QRS features; traditional RLS has a limited suppression effect on 0–5 Hz low-frequency noise; traditional VMD has good performance in suppressing 30–100 Hz high-frequency noise but has the risk of under-decomposition. The improved algorithms show better band selectivity: the improved wavelet performs strong denoising in the high-frequency layer and weak denoising in the low-frequency layer through the hierarchical threshold strategy; the improved RLS adaptively selects the combination of reference signals according to the noise type; the spectral comparison diagram of the improved VMD shows that while completely suppressing the noise bands (0–1 Hz, 30–100 Hz), it almost perfectly retains the energy distribution of the QRS band (5–20 Hz).

The average comprehensive performance indicators of different algorithms are compared under all records of the MIT-BIH Arrhythmia Database, as shown in Table 1.

The improved VMD algorithm proposed in this paper shows significant advantages in multiple core evaluation indicators. As shown in the average values in Table 1, the SNR improvement of the improved VMD reaches 5.17 dB, which is better than all comparative algorithms, reflecting its excellent noise suppression capability. At the same time, the algorithm achieves the lowest MSE (0.2426), PRD (49.13%), and RMSE (0.4925), as well as the highest correlation coefficient (0.875), indicating that while effectively filtering out noise, it better retains the characteristic information of the ECG signal and has high waveform fidelity.

Traditional RLS and improved wavelet algorithms perform well in some indicators, but the SNR improvement of the improved RLS (2.55 dB), although better than its traditional version (2.31 dB), is still significantly lower than that of the improved VMD. It is observed that in complex mixed noise environments, the dependence of RLS-based methods on reference signal quality and linear assumptions may limit the further improvement of their performance.

In terms of computational efficiency, all the compared algorithms achieved Real-Time Processing Ratios (RTR) significantly greater than 1, confirming their fundamental suitability for real-time processing on wearable platforms. Notably, the proposed improved VMD algorithm maintains a processing speed that comfortably exceeds real-time requirements. More importantly, while delivering superior denoising performance, its computational burden remains comparable to that of the traditional VMD, demonstrating that the performance gains are achieved without a substantial increase in complexity. This balance between exceptional signal quality and manageable computational cost makes the improved VMD a practical and efficient solution for resource-constrained wearable devices.

### 3.4. Performance Analysis on Pathological ECG Signals

**Figure 8:**
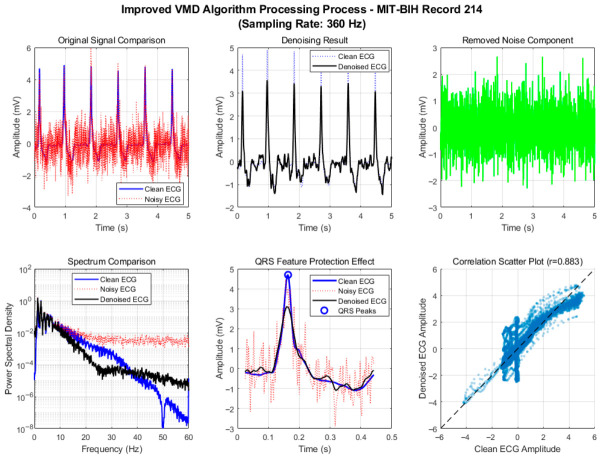
Motion artifact removal process of the improved VMD algorithm on MIT-BIH Record 214 (WPW syndrome).

To evaluate the algorithm’s ability to preserve clinically relevant pathological features, we further tested it on two representative recordings with distinct abnormalities: Record 214 (Wolff–Parkinson–White syndrome, WPW) and Record 217 (third-degree atrioventricular block) from the MIT-BIH Arrhythmia Database. WPW is characterized by a shortened PR interval, widened QRS complex, and the presence of a delta wave—a slurring in the initial upstroke of the QRS complex caused by ventricular pre-excitation. In contrast, complete heart block (Record 217) manifests as a complete dissociation between P waves and QRS complexes, with an escape rhythm producing a broad QRS morphology. Preserving these subtle morphological details during denoising is crucial for accurate diagnosis. Figure 8 illustrates the processing results of the proposed improved VMD on Record 214.

The improved VMD (Figure 8) achieved a high correlation coefficient (0.883) on this record and faithfully preserves the delta wave morphology and the overall QRS width. The sharp features of the delta wave remain clearly visible, and the isoelectric segments are cleaner, aiding in the identification of the pre-excitation pattern. This improvement is attributed to the intelligent IMF selection strategy, which assigns higher weights to components containing QRS band energy and retains those highly correlated with the original signal, thereby protecting diagnostically significant waveform features.

**Figure 9:**
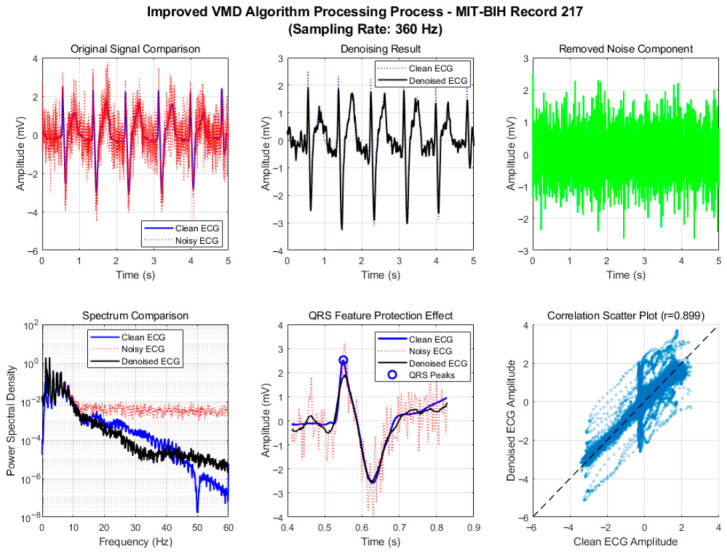
Motion artifact removal process of the improved VMD algorithm on MIT-BIH Record 217 (complete heart block).

For Record 217 (heart block), the results are shown in Figure 9.

The improved VMD (Figure 9) achieves a high correlation coefficient (0.899) and preserves the morphological details of both the QRS complexes and the dissociated P waves. The QRS width and amplitude are faithfully restored, and the isoelectric line is cleaner, allowing for accurate identification of the atrioventricular dissociation pattern. Spectral comparisons indicate that the improved VMD maintains the energy distribution in the QRS band (5–20 Hz) while effectively suppressing out-of-band noise.

**Figure 10:**
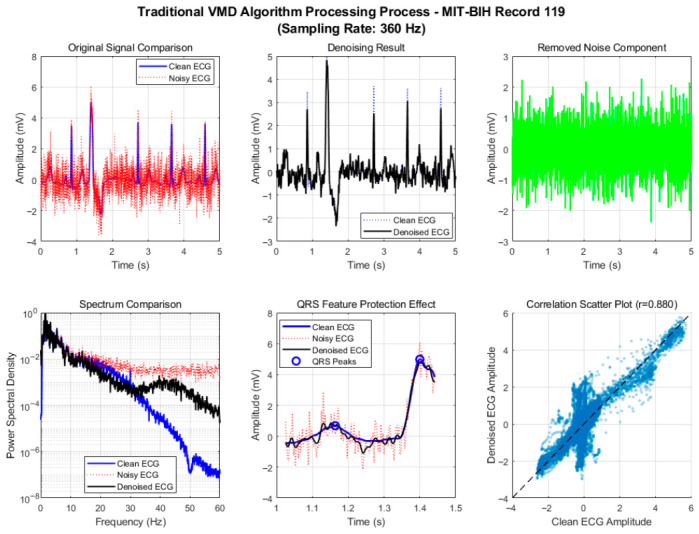
Motion artifact removal process of the traditional VMD algorithm on MIT-BIH Record 119 (premature ventricular contraction).

**Figure 11:**
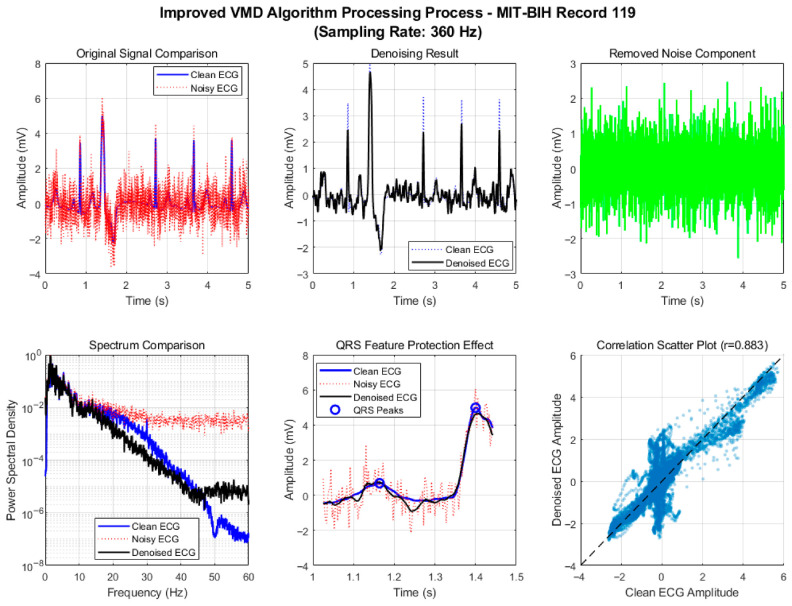
Motion artifact removal process of the improved VMD algorithm on MIT-BIH Record 119 (premature ventricular contraction).

To further verify that the improved VMD does not mistakenly remove pathological beats that may visually resemble motion artifacts, we applied the algorithm to a PVC-containing segment from MIT-BIH Record 119. The traditional VMD (Figure 10) achieves a correlation coefficient of 0.880 on this segment, which is slightly higher than the 0.883 obtained by the improved VMD (Figure 11). The power spectrum of the improved VMD is significantly closer to that of the clean ECG, indicating better preservation of the signal’s frequency content, including the high-frequency components of the PVC. Visual inspection of the QRS region shows that the improved VMD better preserves the morphology of the PVC, with a cleaner isoelectric segment and maintained characteristic width and amplitude, while the traditional VMD introduces more residual noise. These improvements are attributed to the intelligent IMF selection strategy, which assigns higher weights to components containing QRS-band energy (5–20 Hz) and retains those highly correlated with the original signal. Even for an abnormal beat like a PVC, its steep slopes and dominant frequency content still fall within the QRS band, and its correlation with the original ECG remains high. Therefore, the algorithm does not mistakenly remove the PVC as a motion artifact. In contrast, motion artifacts typically have most of their energy below 5 Hz and exhibit lower kurtosis, making them distinguishable by the multi-feature scoring system.

The above case studies demonstrate that the improved VMD algorithm not only removes motion artifacts effectively but also preserves clinically significant pathological features, including low-amplitude P waves and subtle delta waves. The multi-feature IMF scoring mechanism, which gives high weight to spectral characteristics in the QRS band and considers correlation with the original signal, ensures that components containing diagnostically critical information are retained. The superior quantitative metrics of the proposed algorithm, combined with the visual preservation of key diagnostic features, confirm that the proposed adaptive VMD framework excels not only in general denoising tasks but also in maintaining pathological ECG characteristics. This is crucial for wearable ECG monitoring devices aiming for reliable clinical applications.

### 3.5. Parameter Optimization and Validation via Deep Learning-Based Arrhythmia Classification

To further validate the proposed adaptive VMD framework and to optimize its key parameters (number of modes K and feature weights in Equation (9)), a systematic evaluation was conducted using a deep learning-based arrhythmia classification pipeline. This pipeline directly measures the impact of the denoising algorithm on a clinically relevant task—automatic classification of heartbeats into multiple arrhythmia types—and provides quantitative guidance for parameter selection.

#### 3.5.1. Classification Pipeline

**Figure 12:**
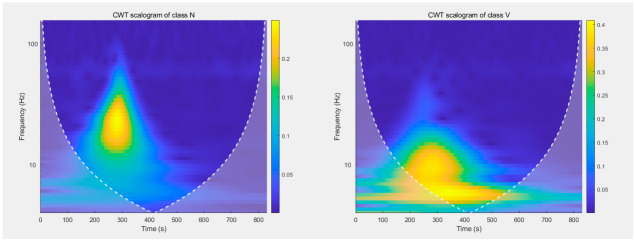

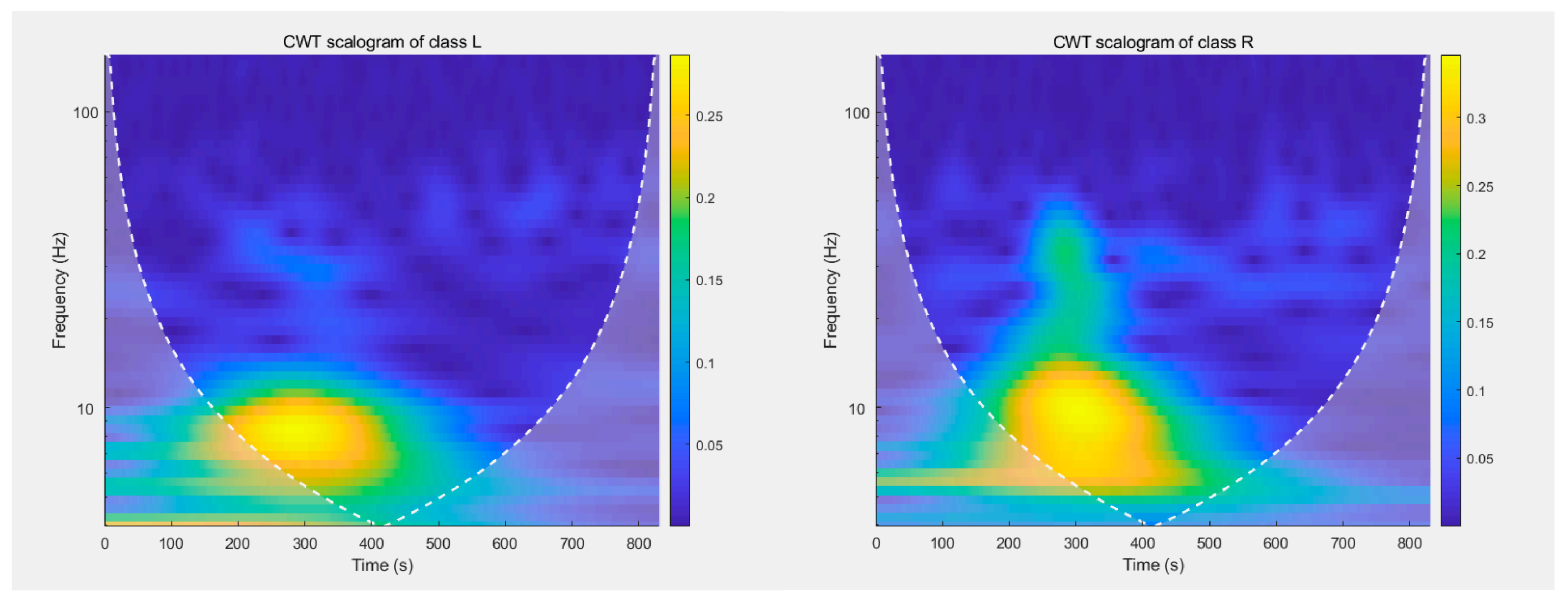
Images of Class L, Class N, Class V and Class R under CWT transformation.

**Figure 13:**
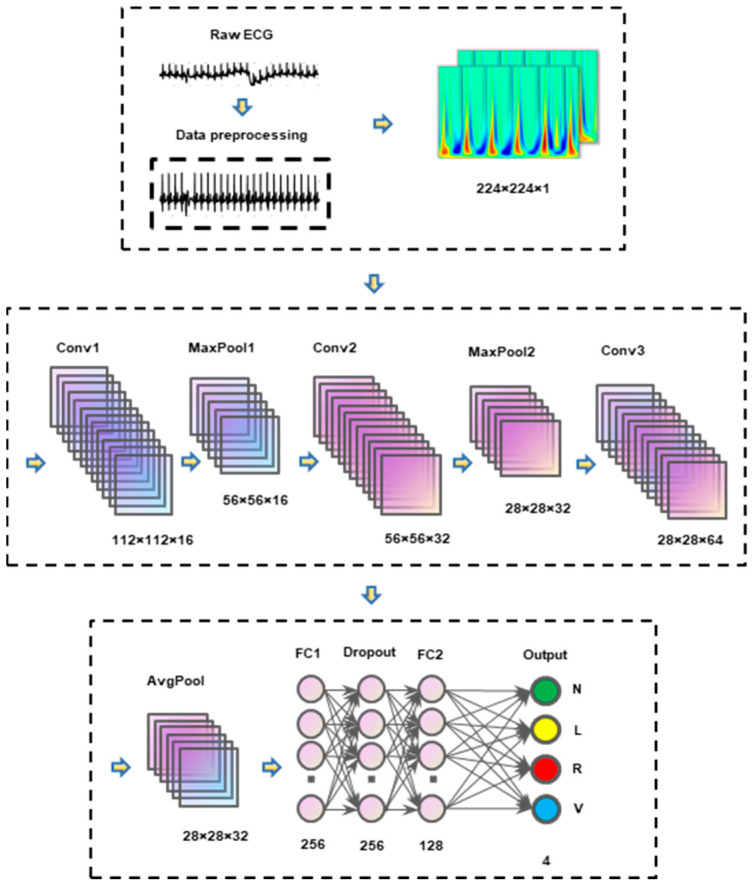
Structure diagram of the CNN model for arrhythmia classification.

The classification pipeline consists of four stages:

1. Denoising: Improved VMD is applied to full-length ECG signals from the MIT-BIH Arrhythmia Database using different K and weight configurations.
2. Heartbeat extraction: Based on annotated R-peak positions, heartbeats are extracted with a window of 100 samples before the R-peak and 199 samples after (total length 300 samples, sampling rate 360 Hz).
3. Time-frequency transformation: Continuous wavelet transform (CWT) is applied to each heartbeat to generate scalogram images [26]. Figure 12 shows representative CWT scalograms of four heartbeat classes (N, V, L, R) from the MIT-BIH database. These color images capture both time and frequency characteristics of the ECG signal, providing rich input features for the deep learning classifier.
4. Deep learning classification: A convolutional neural network (CNN) is trained to classify heartbeats into four AAMI-recommended classes: N (normal), V (ventricular ectopic), L (left bundle branch block), and R (right bundle branch block). The four types of CWT images for different categories of arrhythmias are shown in Figure 12. The architecture of the CNN is illustrated in Figure 13 [27], and it is detailed in Table 2.

To capture the frequency distribution of ECG signals and fully leverage the powerful visual feature extraction capability of CNN, DWT converts the non-stationary time-series signals of four heartbeat classes (N, V, L, R) into the two-dimensional time-frequency maps shown in Figure 12.

The CNN architecture processes 224 × 224 × 1 grayscale images—specifically, the CWT scalograms of ECG heartbeats. The network begins with an input layer that accepts these images. It then passes through three convolutional layers: the first uses a 3 × 3 filter with 16 output channels, a stride of 2, and padding to maintain spatial dimensions, followed by a ReLU activation function and a 2 × 2 max-pooling layer; the second uses a 3 × 3 filter with 32 output channels and “same” padding, followed by ReLU and another 2 × 2 max-pooling layer; and the third uses a 3 × 3 filter with 64 output channels and “same” padding, followed by ReLU and a 7 × 7 average pooling layer. These layers progressively reduce the spatial dimensions while increasing the depth of the feature maps. The output from the convolutional layers is then flattened and passed through two fully connected layers: the first with 128 neurons followed by ReLU and a dropout layer with a rate of 0.3 to prevent overfitting, and the second with 64 neurons followed by ReLU. The final fully connected layer has 4 neurons corresponding to the four classes of ECG signals, and it uses a softmax activation function to output class probabilities. Training uses the Adam optimizer (initial learning rate 1 × $10^{-4}$), mini-batch size 32, and 20 epochs, with a 70%/15%/15% split for training, validation, and testing.

#### 3.5.2. Experimental Design

To simulate the short data blocks typical of wearable devices, each signal segment was fixed to N = 1000 samples. Three candidate numbers of modes were tested: K = 4, 6, 8. For each K, three weight vectors for the four features (QRS band power ratio, noise band power ratio, correlation with original signal, and kurtosis) were evaluated:

(1) W1 = [0.4, 0.3, 0.2, 0.1], derived by the analytic hierarchy process (AHP) as described in Section 2.2.5;

(2) W2 = [0.25, 0.25, 0.25, 0.25], uniform weights;

(3) W3 = [0.1, 0.2, 0.3, 0.4], reversed importance order.

Raw (non-denoised) signals were also classified as a baseline. To avoid overfitting and ensure balanced evaluation, each class was limited to 1000 randomly selected heartbeats. The same random seed was used for all configurations to ensure fair comparison.

#### 3.5.3. Results and Analysis

**Figure 14:**
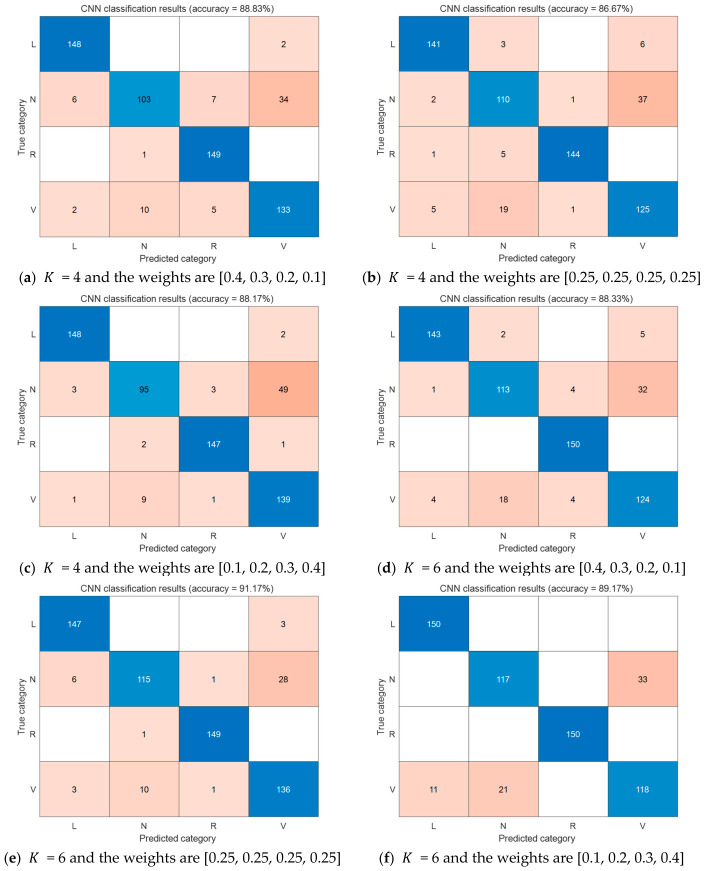

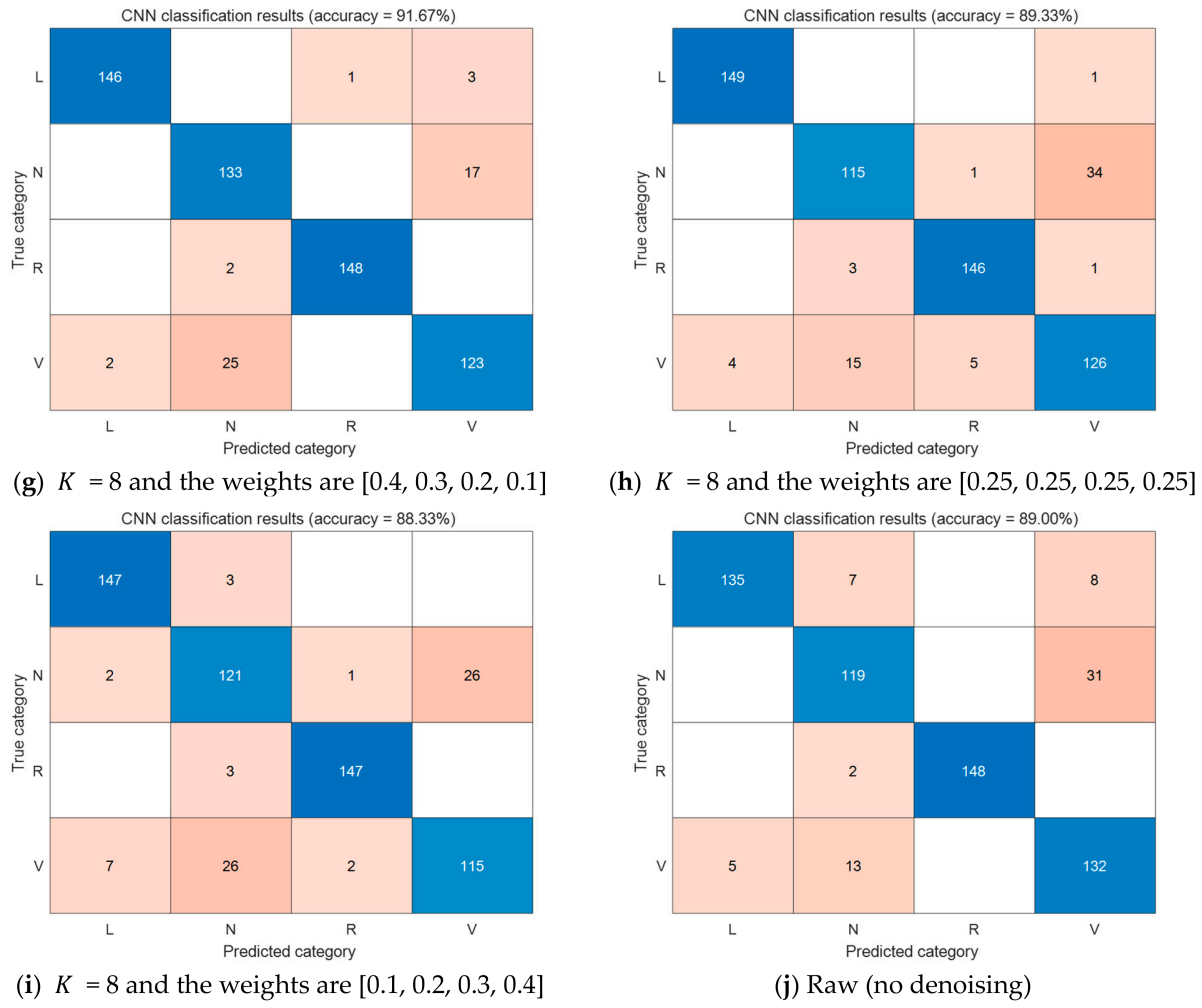
Test accuracy of the CNN-based arrhythmia classifier on the MIT-BIH dataset under different VMD parameter configurations. (**a**–**i**) Results for K = 4, 6, 8 with different weight vectors; (**j**) baseline result on raw (non-denoised) signals.

Figure 14 shows the test accuracy of the CNN-based arrhythmia classifier under different values of K and different weight settings.

The accuracy values presented in Figure 14 provide a visual comparison of classification performance across different parameter settings. To facilitate a more detailed quantitative analysis, Table 3 summarizes the exact accuracy percentages corresponding to each configuration shown in Figure 14, along with the baseline result for raw, non-denoised signals.

The results from Figure 14 and Table 3 reveal several important findings.

First, the proposed denoising framework substantially improves deep learning-based arrhythmia classification. The best configuration, i.e., K = 8 with the AHP-derived weight vector W1 = [0.4, 0.3, 0.2, 0.1], achieves a test accuracy of 91.67%. This represents a 2.67 percentage point improvement over the raw signal baseline (89.00%), confirming that effective motion artifact removal enhances the discriminability of heartbeat features and directly benefits automatic arrhythmia diagnosis.

Second, the effect of the number of modes K is not monotonic. Among the three K values tested, the highest overall accuracy (91.67%) is obtained with K = 8 and W1. However, when using the uniform weight vector W2 = [0.25, 0.25, 0.25, 0.25], K = 6 yields an accuracy of 91.17%, which is higher than the 89.33% achieved with K = 8. This observation suggests that the optimal number of modes depends on the weighting scheme, and that a larger K does not always guarantee better performance.

Third, the AHP-derived weight vector W1 consistently outperforms the reversed vector W3 = [0.1, 0.2, 0.3, 0.4] across all K values. For instance, with K = 8, W1 achieves 91.67% compared to 88.33% for W3; with K = 6, W1 achieves 88.33% compared to 89.17% for W3 (though the difference is smaller); and with K = 4, W1 achieves 88.83% compared to 88.17% for W3. These results validate the relative importance of spectral features—particularly the QRS band power ratio—over kurtosis, and confirm that the AHP-based weight assignment is superior to heuristic or equal weighting strategies.

In summary, the systematic parameter optimization and deep learning-based classification validation provide strong quantitative evidence that the proposed adaptive VMD framework achieves superior denoising performance (as shown in Section 3.3 and Section 3.4) and delivers tangible improvements in a clinically relevant task—arrhythmia classification. The optimal parameters identified (K = 8 and weights [0.4, 0.3, 0.2, 0.1]) are therefore recommended for wearable ECG monitoring applications.

## 4. Conclusions

Aiming at the problem of motion artifact interference faced by wearable ECG monitoring devices in dynamic environments, this paper proposes an adaptive denoising framework based on improved Variational Mode Decomposition (VMD), providing an effective technical approach for improving the quality of dynamic ECG signals [28].

The main innovations are reflected in the following three aspects: first, a data-adaptive VMD parameter adjustment mechanism is designed to dynamically optimize the number of modes K and bandwidth parameter α according to the input signal length, overcoming the limitation of traditional methods relying on experience for parameter setting; second, a multi-feature fusion-based intelligent IMF selection strategy is constructed to score and screen the decomposed Intrinsic Mode Functions by comprehensively evaluating their spectral, statistical, and correlation features, realizing accurate separation of signal and noise components; third, a systematic performance comparison and verification system is established, conducting comprehensive comparisons with five traditional and improved algorithms including wavelet, RLS, and VMD, and evaluating the algorithm performance from multiple dimensions such as denoising effect, waveform preservation, and real-time performance.

Experimental results on the public MIT-BIH Arrhythmia Database show that the improved VMD algorithm has the best overall performance in key indicators: SNR improvement reaches 5.17 dB; MSE, PRD, and RMSE are the lowest among similar algorithms; and the correlation coefficient and PSNR are the highest. In addition, the algorithm has good real-time performance (RTR = 22.5), which can meet the low-power consumption and real-time processing requirements of wearable devices. Furthermore, analysis on pathological cases (Wolff–Parkinson–White syndrome and complete heart block) demonstrates that the improved VMD retains critical diagnostic morphologies (e.g., delta waves and P waves) effectively, confirming its suitability for wearable devices aimed at clinical monitoring [29]. To further validate the clinical utility of the proposed denoising framework, it was integrated into an end-to-end arrhythmia classification pipeline based on continuous wavelet transform (CWT) and convolutional neural network (CNN). Systematic parameter optimization over different numbers of modes K and feature weight configurations showed that the best classification performance (91.67% accuracy) is achieved with K = 8 and the AHP-derived weights [0.4, 0.3, 0.2, 0.1], which is 2.67 percentage points higher than the raw signal baseline (89.00%). This confirms that the improved VMD not only removes motion artifacts but also preserves diagnostically critical features, thereby boosting the performance of subsequent AI-based diagnostic models [30]. In summary, the improved VMD algorithm proposed in this study achieves a good balance between denoising performance and computational efficiency while preserving clinically relevant pathological features, providing a feasible signal preprocessing solution that enables wearable ECG devices to achieve high-precision dynamic monitoring. Future research will further verify the algorithm’s robustness on more diverse pathological ECG signals (e.g., those with pronounced ST-segment deviations, ventricular fibrillation, or other complex arrhythmias) to assess its clinical applicability.

## Figures and Tables

**Table 1:** Performance comparison of different denoising algorithms under simulated motion artifacts (SNR = 1 dB).

Algorithm	SNR Improvement (dB)	MSE	PRD (%)	Correlation Coefficient R	RMSE	Processing Time (s)	RTR
Traditional Wavelet	1.08	0.6214	78.69	0.621	0.7883	0.007	4784.0
Traditional RLS	2.32	0.4693	68.35	0.823	0.6851	0.029	1993.1
Traditional VMD	4.29	0.2951	54.38	0.847	0.5432	2.313	22.5
Improved Wavelet	4.14	0.3067	55.38	0.834	0.5538	0.014	3714.9
Improved RLS	2.55	0.4478	66.64	0.796	0.6692	0.062	913.3
Improved VMD	5.17	0.2426	49.13	0.875	0.4925	2.359	22.5

**Table 2:** Architecture of the convolutional neural network.

Layer	Type	Parameters	Output Size
Input	Image Input Layer	224 × 224 × 1	224 × 224 × 1
Conv1 + ReLU + Pool	Conv(3 × 3, 16) + MaxPool(2)	Stride = 2, Padding = 1	56 × 56 × 16
Conv2 + ReLU + Pool	Conv(3 × 3, 32) + MaxPool(2)	Padding = same	28 × 28 × 32
Conv3 + ReLU + AvgP	Conv(3 × 3, 64) + AvgPool(7)	Padding = same	4 × 4 × 64
FC1 + Dropout	Fully Connected + Dropout	128 neurons, Dropout 0.3	128
FC2	Fully Connected	64 neurons	64
Output	Fully Connected + Softmax	4 neurons	4 (class probs)

**Table 3:** Classification accuracy (%) of the CWT-CNN pipeline on MIT-BIH heartbeats (N, V, L, R classes) after denoising with different VMD parameter settings. Raw (non-denoised) signals serve as baseline.

K	Weights	Accuracy (%)
4	[0.4, 0.3, 0.2, 0.1]	88.83
4	[0.25, 0.25, 0.25, 0.25]	86.67
4	[0.1, 0.2, 0.3, 0.4]	88.17
6	[0.4, 0.3, 0.2, 0.1]	88.33
6	[0.25, 0.25, 0.25, 0.25]	91.17
6	[0.1, 0.2, 0.3, 0.4]	89.17
8	[0.4, 0.3, 0.2, 0.1]	91.67
8	[0.25, 0.25, 0.25, 0.25]	89.33
8	[0.1, 0.2, 0.3, 0.4]	88.33
Raw (no denoising)	/	89.00

## Data Availability

The raw data supporting the conclusions of this article will be made available by the authors on request. The original data presented in the study are openly available in the MIT-BIH Arrhythmia Database at https://physionet.org/content/mitdb/1.0.0/ (accessed on 24 April 2026).
